# Solving the missing heritability problem

**DOI:** 10.1371/journal.pgen.1008222

**Published:** 2019-06-24

**Authors:** Alexander I. Young

**Affiliations:** Big Data Institute, University of Oxford, Oxford, United Kingdom; University of California Los Angeles, UNITED STATES

The problem of missing heritability, that is to say the gap between heritability estimates from genotype data and heritability estimates from twin data, has been a source of debate for about a decade [1]. It might appear that the advent of whole genome sequence data on tens of thousands of people is poised to resolve the issue, but here I want to sound a note of caution: more sequence data does not mean methodological problems go away…

Heritability measures the overall importance of genetic inheritance in shaping differences between individuals and is defined as the fraction of trait variation in a population due to genetic inheritance [2]. The advent of twin studies[3] made it possible to estimate heritability by comparing the phenotypic similarity of identical (monozygotic) twins to non-identical (dizygotic) twins: since monozygotic twins are genetically identical, whereas non-identical twins are only half identical on average, greater similarity of identical over non-identical twins is evidence for a contribution of genetic variation to trait variation. However, the twin design makes several assumptions, most importantly that there is no greater environmental similarity of identical over non-identical twins. Whether twin studies have overestimated heritability for human traits, especially social and behavioural traits, remains controversial [4].

The dawn of the genome-wide association study (GWAS) era, around the year 2007, brought with it the question: can we identify specific genetic variations that explain the heritability estimated from twin studies? The small sample sizes of early GWAS meant they had power to identify only common genetic variants with relatively strong effects, and the amount of trait variation that these variants explained was typically only a small fraction of the heritability estimated by twin studies. For height, by 2010 around 40 variants had been identified that collectively explained around 5% of the variation in height, compared to a twin heritability of around 80% [5]. This gap became labelled 'the problem of missing heritability' and has stimulated heated debate ever since [1].

Many different explanations for the 'missing heritability' have been proposed [6]. I will focus on two: 1) that complex traits are highly polygenic and affected by many rare variants; 2) that twin studies have overestimated heritability. Note that both of these explanations could contribute to explaining the ‘missing heritability’. The idea behind 1) was that GWAS were not sufficiently powerful to detect the many genetic variants with weak effects on a trait like height, and the genotyping array technologies were not capturing the rare genetic variants that may explain a substantial fraction of the heritability [1,5,6]. The idea behind 2) was that twin studies were overestimating heritability, perhaps due to genetic interactions [7], gene-environment interactions[8], or violation of twin studies assumptions about the environment [4], so that less heritability was in fact missing.

The deepest solution to the missing heritability problem would involve identifying all of the causal genetic variants and measuring how much trait variation they explain. An intermediate step towards this solution is to show how much variation we could hope to explain from all measured genetic variation, even if we do not have the statistical power to identify all of the specific causal variants.

In 2010, a paper took a step towards this intermediate solution by showing that ~45% of the variation in height could be explained by the genetic variation captured on a particular genotyping array that measured ~250k common single nucleotide polymorphisms (SNPs) (Table 1). This implied that the genetic variation captured on the genotyping array explained a lot more of the variation in height than the particular variants that had been identified to affect height at the time. Therefore, there were many common variants with relatively weak effects on height that had been missed by GWAS due to a lack of statistical power.

**Table 1:** Heritability of height estimated by different methods.

Genetic data type	Method	Population	Estimate	S.E.	Reference
None	ACE (Twins)	European (various)	0.73–0.81	-	Silventoinen et al. 2003 [29]
SNP array	GREML	Australian	0.45	0.08	Yang et al. 2010 [5]
SNP array + imputation	GREML-LDMS	European ancestry meta-analysis	0.56	0.023	Yang et al. 2015 [12]
Whole genome sequence	GREML-WGS	European ancestry (USA)	0.79	0.09	Wainschtein et al. 2019 [13]
Identity-by-descent sharing	RDR	Iceland	0.55	0.045	Young et al. 2018 [27]
Identity-by-descent sharing	Sib-Regression	European ancestry meta-analysis	0.68	0.079	Young et al. 2018[27] and Hemani et al. 2013 [30].

We give the range of sex-averaged estimates of the heritability of height from the ACE model from European ancestry samples in seven different countries [26]. The other estimates are taken from main results of the referenced papers, apart from the Sib-Regression estimate, which is a fixed-effects meta-analysis estimate combining the estimate from Iceland [27] and from a previous meta-analysis that did not include Icelandic data [28].

The authors employed a methodology that later became termed GREML (Genomic Relatedness Restricted Maximum Likelihood) [9,10]. The GREML methodology estimates the variance explained by the SNPs by measuring how phenotypic similarity changes with SNP similarity. Typically, GREML restricts the analysis to distantly related individuals in order to avoid bias due to certain kinds of environmental effects shared between close relatives and genetic interactions [5,10]. The GREML methodology can only capture phenotypic variation explained by SNPs that are correlated with genotyped SNPs due to linkage disequilibrium (LD) [9–11]. Genotyping arrays mostly measure genetic variants that are common in the population, and most rare variants are in low LD with the common variants on a typical genotyping array [12,13], so the GREML methodology applied in 2010 was unable to capture most of the phenotypic variation explained by rare variants. In 2015, the GREML methodology was extended to include rarer genetic variations inferred by imputation [12], a statistical procedure that can infer genetic variants not measured on a genotyping array through reference to more complete genome sequence data. This increased the variance explained for height from 45% to 56% (Table 1). The question then remained: was the ~80% number from twin studies too high, or do very rare variants that cannot be imputed accurately explain the gap?

One approach to answering this question is to extend the GREML methodology to high quality whole genome sequence (WGS) data[13], an extension that I’ll call GREML-WGS. WGS data directly measures all genetic variants. If GREML-WGS could show that the variance explained by all sequence variants was in line with twin heritability estimates, this would suggest that the full twin heritability is waiting to be unlocked by large samples with whole genome sequence data. Initial results suggest that a substantial fraction of height variation is explained by the effects of very rare variants that are not well imputed[13]. This result is plausible for traits under selection, which will tend to make alleles with large effects on traits rare in the population[14]. If the gap between heritability estimated from imputed SNPs and twin heritability is accounted for by the effects of rare variants that are not well imputed, this would be an important step towards solving the missing heritability problems and be informative of the genetic architecture of complex traits. I therefore outline a series of challenges that would need to overcome before we could be confident of such a result from GREML-WGS.

The first challenge is one of precision. The information used to estimate heritability from rare variants by GREML-WGS comes from the variation in sharing of rare variants among distantly related pairs of individuals [13, 15]. However, distantly related individuals typically do not share any particular rare variant, so the variation in rare variant sharing is low. This means that large samples with high quality WGS data are required to obtain precise estimates, and such samples are not common yet. Based on the only existing application of GREML-WGS [13], a sample size of ~40,000 would produce estimates precise enough to be statistically distinguished from other heritability estimates (Table 1). It is likely that this challenge will be overcome shortly, since samples of similar magnitude already exist [16].

However, when the challenge of achieving sufficient precision of GREML-WGS estimates is overcome, questions about methodological assumptions remain. The methodology assumes that effect sizes are normally distributed within each bin, where the variants have been divided into bins based upon their frequency and the strength of their correlations with other variants (LD). Since GREML makes inferences about the distribution of effect sizes, GREML heritability estimates can become biased when assumptions about the distribution of effect sizes are violated [17]. This could be more problematic for the rare variants used in GREML-WGS than for the common variants used in standard GREML, as one expects there to be a small fraction of rare variants with strong effects, implying a large deviation from the assumed normal distribution of effect sizes.

Population stratification presents another challenge for the GREML-WGS methodology. Population stratification occurs when two genetically distinct subpopulations have different mean trait values. This implies that any genetic variant that is differentiated between these subpopulations (which is usually due to chance, i.e. genetic drift) will be correlated with the trait even though it has no causal effect on the trait. Recently, two papers were published showing that stratification has affected genome-wide association studies of height, leading to spurious inferences about selection on height in Europe [18–20]. This work has shown that stratification can remain problematic even after attempting to correct for it using principal component analysis (PCA) [21], a technique that attempts to infer the major axes of genetic variation in a population (principal components), which are typically associated with geographic separation [22].

If mean trait values differ along the major principal components, adjusting for the major principal components can remove bias due to population stratification. However, it is hard to accurately infer all of the relevant axes of genetic variation that may be correlated with mean trait values in order to completely eliminate bias due to stratification [23]. The situation is even trickier for the rare variants used in GREML-WGS. Rare variants tend to have more complicated spatial distributions than common variants, making it even more difficult to infer the axes of genetic variation required to remove bias due to stratification [24, 25].

The linear mixed model methodology underlying GREML methods can also be used to adjust for stratification in GWAS [30–32]. Linear mixed model GWAS methods model the effects of genome-wide SNPs jointly with the focal SNP, resulting in an estimate of the variance explained by the genome-wide SNPs and an estimate of the effect of the focal SNP. Linear mixed model GWAS can account for more complicated patterns of stratification than PCA by modelling the effects of all genome-wide SNPs, rather than considering stratification along the major principal components alone, leading to reduced bias in SNP effect estimates compared to PCA adjustment [32]. However, this implies that those stratification effects that linear mixed models pick up, but PCA misses, are likely to contribute to the heritability estimate from GREML, leading to an overestimation of heritability. Supporting this, I have found evidence that the heritability of height in Iceland was overestimated by a method that is very similar to GREML [27], and I suspect that this overestimation was due in part to population stratification that had not been properly controlled for by PCA.

The problem of population stratification is even trickier for the very rare variants used by GREML-WGS. The GREML-WGS methodology measures the contribution from rare variants in part by measuring the degree to which pairs of individuals who share rare variants tend to have more similar phenotypes than people who do not. However, if a pair of individuals share a very rare variant, then it is likely that they inherited this variant from a recent common ancestor, even if their genome-wide relatedness is low. Pairs of individuals who share a recent common ancestor are more likely to have similar environments than those who do not, implying that the GREML methodology could mistakenly infer contributions from rare genetic variants that are in fact environmental contributions. It is hard to see how this type of stratification could be corrected for by PCA because it is specific to particular pairs or clusters of individuals who share a recent common ancestor.

It will be difficult to assess the impact of population stratification on GREML-WGS without using some form of family data, where the randomisation of genetic material during meiosis can be used to disentangle genetic from environmental influences [34–37]. Family data can also be used to estimate heritability in a way that is robust to population stratification. Siblings vary in their relatedness due to random inheritance of the same or different copies of parental chromosomes. A method that I call Sib-Regression takes advantage of the random variation in relatedness between siblings in a family to estimate heritability with little bias from population stratification and environment [38]. However, Sib-Regression requires hundreds of thousands of genotyped siblings pairs to obtain precise estimates. Last year, I described a method, relatedness disequilibrium regression (RDR), that generalises Sib-Regression to all relative pair classes, gaining precision while retaining robustness to population stratification [27]. Ideally, one would obtain WGS data on a large sample of families and compare Sib-Regression, RDR, and GREML-WGS estimates.

**Fig 1:**
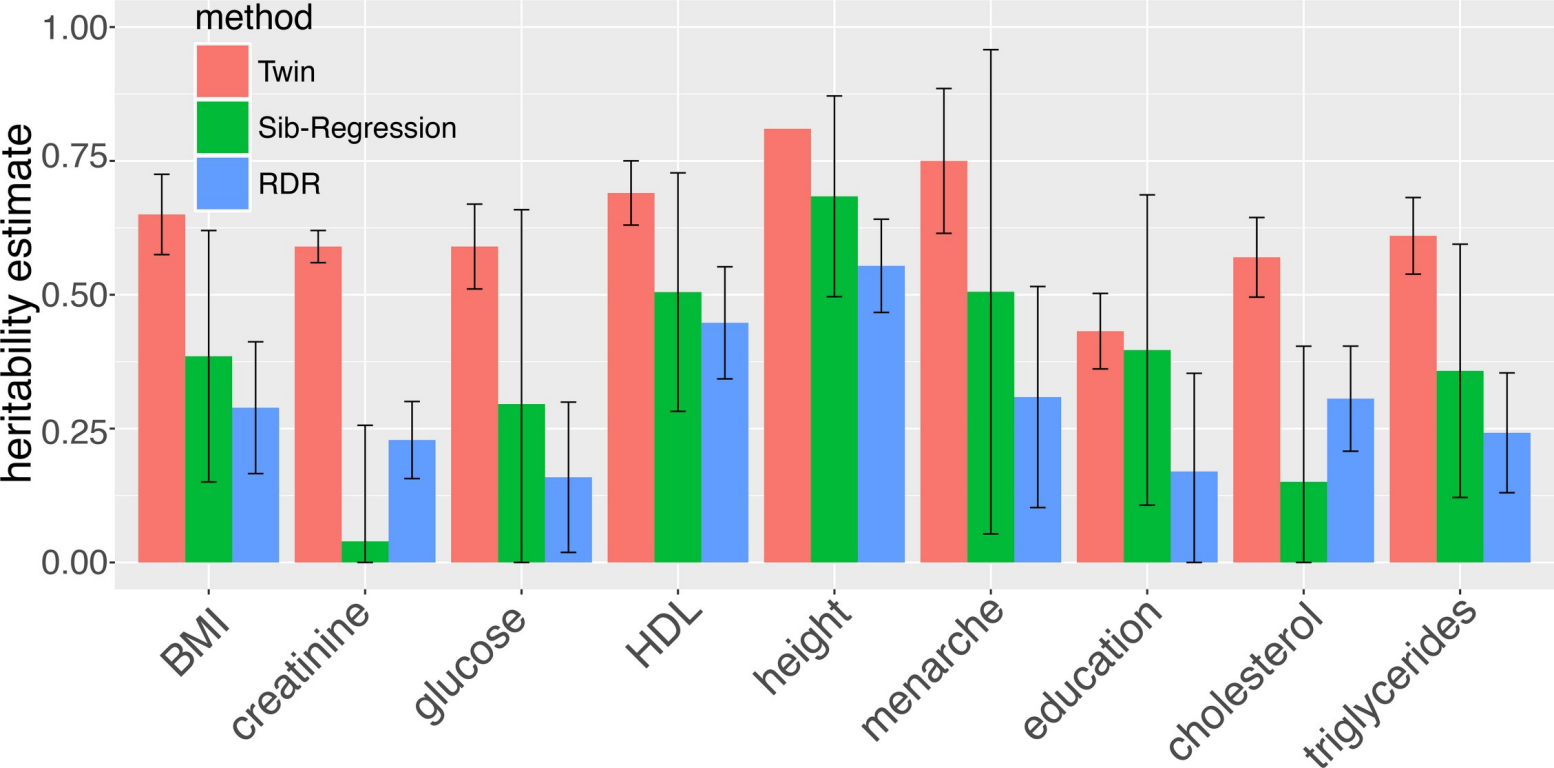
Comparison of heritability estimates from RDR and Sib-Regression in Iceland to Swedish twin studies. The error bars give 95% confidence intervals for the estimates. The estimates are taken from Young et al. 2018 [27]. The RDR and Sib-Regression estimates are from Icelandic samples, and the Swedish twin estimates are taken from various publications utilising the Swedish twin registry [27,33].

We can examine existing heritability estimates from RDR and Sib-Regression (Table 1) to get a sense of what we might expect from precise GREML-WGS estimates. The RDR estimate of the heritability of height in Iceland is 55% (S.E. 4.4%). The Sib-Regression estimate is 68% (S.E. 9.6%), which gives an estimate of 68% (S.E. 7.9%) when combined with a previous estimate [30]. These estimates suggest that the heritability of height may be lower than estimated by twin studies. Furthermore, if one compares RDR and Sib-Regression estimates to twin estimates, the RDR and Sib-Regression estimates are consistently lower across traits (Fig 1). This implies that even with WGS data there may still be some ‘missing heritability', in that there is still a gap between heritability estimated from robust genomic methods (RDR and Sib-Regression) and twin estimates.

While my methodological concerns about GREML-WGS might be answered through further analyses for a trait like height, my own work has shown that the GREML approach leads to substantial overestimation of heritability for traits like educational attainment [27]. This is due to the influence of indirect genetic effects (‘genetic nurture’) from relatives [35], which are the effects of genetic variants in relatives (mostly siblings and parents) on an individual through their environment. Family data is required to adjust for indirect genetic effects from relatives. Therefore, solving the problem of missing heritability for traits like educational attainment will require large samples of families with WGS data.

Further collection of family data may also contribute to solving a related puzzle about genetic prediction. By using the estimated effects of genome-wide SNPs, a model that predicts trait values from genotype data can be constructed, termed a polygenic score [38]. While the correlation between the polygenic score and educational attainment suggests that it can predict around 11–13% of the variation in educational attainment, within-family analyses suggest that at least half of this predictive ability comes from indirect genetic effects from relatives, population stratification, and assortative mating [35, 39]. Similar results have been obtained for other cognitive and behavioural traits [40, 41]. The within-family design removes the total influence of indirect genetic effects from relatives, assortative mating, and population stratification; however, the relative contribution of these different factors to polygenic prediction is not well characterised. Building a better understanding of assortative mating and the relationship between genetic and environmental influences on traits will form a key part of the deep solution to the missing heritability problem, which will also leverage whole genome sequence data to construct a more detailed understanding of genetic architecture and stronger polygenic predictors.
